# Nanoemulsions containing *Garcinia mangostana* L. pericarp extract for topical applications: Development, characterization, and *in vitro* percutaneous penetration assay

**DOI:** 10.1371/journal.pone.0261792

**Published:** 2021-12-23

**Authors:** Elsa Anisa Krisanti, Dyah Paramawidya Kirana, Kamarza Mulia

**Affiliations:** Department of Chemical Engineering, Universitas Indonesia, Depok, Indonesia; Institute for Biological Research, University of Belgrade, SERBIA

## Abstract

A highly stable oil-in-water nanoemulsion for topical applications, containing mangostins extracted from the pericarp of mangosteen (*Garcinia mangostana L*.), is a promising strategy to protect mangostins as well as to improve penetration of these important antioxidants through the skins. Nanoemulsions consisted of virgin coconut oil as the oil phase, Tween-80 and Span-80 as surfactants, and xanthan gum as the thickening agent, were prepared using the high-energy and low-energy emulsification methods. The nanoemulsions that were stable up to 28 days had oil droplet diameter of 220 nm to 353 nm and zeta potential of -46.9 mV to -63.7 mV. The accelerated stability test showed that the most stable nanoemulsions were those prepared using the low-energy emulsification method with an estimated shelf life of eleven months, composed of 11% oil phase, 17% surfactant, and 72% aqueous phase. The *in vitro* percutaneous penetration test for the nanoemulsion with added xanthan gum provided high cumulative skin penetration of mangostins of up to 114 μg/cm^2^. The results of this study indicate that virgin coconut oil-based nanoemulsions containing mangostins, prepared using the low-energy emulsification method, stabilized by xanthan gum and mixed at 40°C can prospectively be used for topical applications.

## Introduction

The topical application of extracts containing antioxidant bioactives, such as mangostins and other xanthones, have been hindered because these highly lipophilic bioactives have low solubilities in water and they are susceptible to degradation under normal conditions. Emulsion-based delivery systems are particularly suited for the encapsulation of mangosteen extract within an oil-in-water (o/w) nanoemulsion [1], having a small droplet diameter of less than 200 nm [2]. Nanoemulsion has become an effective approach to protect and improve dispersability of bioactives in aqueous solutions, which can increase percutaneous absorption in topical application. Nanoemulsion have several advantages over conventional emulsions, such as the increased bioavailability of encapsulated bioactives, high stability against droplet aggregation and separation, and increased shelf life for commercial products [3].

The water phase of the nanoemulsions increases the hydration of the top skin layer (stratum corneum), which leads to a rapid increase in permeability and then a slowdown to a steady state diffusion [4]. In effective topical applications, the bioactives are able to diffuse through the skin, depending upon several factors: the solubility and diffusivity of the bioactives, the oil or water partition coefficient, the concentration of the bioactive, the surface area of the skin, and the thickness of the top skin layer [5]. Nanoemulsions can maximize the penetration of drugs and bioactives into the skin [6], form a film on the skin, as well as having high stability and pleasant aesthetic characteristics and skin feelings [7, 8].

In this study, mangostins and other xanthones were extracted from the pericarp of mangosteen fruit (*G*. *mangostana*). Mangosteen grows in the tropical rainforests of southeast Asian countries, such as Indonesia and Malaysia. The pericarp of mangosteen has been traditionally used for the treatment of abdominal pain, diarrhea, eczema, and chronic ulcers. Recent studies show that the extract of this pericarp has numerous pharmaceutical activities, such as anti-inflammatory, cytotoxic, antioxidant, antitumoral, immunomodulatory, neuroprotective, antiallergic, antibacterial, and antiviral properties [9, 10].

Nanoemulsions could be prepared using a high-shear stirring emulsification or a spontaneous emulsification method referred to as the high-energy (HE) and the low-energy (LE) emulsification method, respectively. The HE method requires a mechanical device that can combine the oil and water phases and break down oil droplets. On the other hand, the LE method relies on oil or water droplets’ physicochemical properties to form emulsions spontaneously when in contact with a combination of surfactants. This method is widely developed and used due to its simplicity, formation ease, and cost-effectiveness [11].

The emulsification of active compounds using vegetable oils has been reported for cosmetic applications [12]. It has been known that virgin coconut oil (VCO) increases the moisture and improves moisture-locking barrier of the skin. The fatty acids contained also have antibacterial effects, disrupting cell membrane of *Clostridium difficile* [13]. The solubilities of the mangosteen extract in VCO, palm kernel oil (PKO), and extra virgin olive oil (EVOO) were determined and the vegetable oil that provided the highest solubility was used as the oil phase. Tween 80 and Span 80 were mixed in specific molar ratios to obtain surfactants with the proper hydrophilic lipophilic balance (HLB), and thus, form the most stable nanoemulsion. Xanthan gum, a thickening agent which consists of repeating units of pentasaccharide, was used as the nanoemulsion stabilizer due to its elastic properties. Xanthan gum is stable in both alkaline and acidic conditions and it has the best thermal stability among the water-soluble hydrocolloids [14].

The purpose of this research was to obtain highly stable nanoemulsions for topical applications that contain high amounts of mangostins extracted from the pericarp of mangosteen fruit, and, to determine the physicochemical characteristics of the nanoemulsions (appearance and organoleptic, pH, viscosity, droplet size, zeta potential, antioxidant activity, and skin penetration ability).

## Materials and methods

### Materials

Mangosteen pericarp was obtained from Solo, central Java, Indonesia, and identified as *G*. *mangostana* by Herbarium Bogoriense, Research Center for Biotechnology, Indonesian Institute of Sciences (LIPI). Analytical grade ethanol (96%), ethyl acetate, sodium biphosphate, potassium biphosphate, and xanthan gum were purchased from Merck. Span 80 and Tween 80 were purchased from Asian Chemicals. Quercetin (95%) and DPPH or 2,2-diphenyl-1-picrylhydrazyl (95%) were purchased from Sigma Aldrich. Virgin coconut oil was purchased from Nucifera Indonesia, palm kernel oil purchased from Cortico Mulia Sejahtera Indonesia, and extra-virgin olive oil was purchased under the brand name Borges.

### Extraction of mangosteen pericarp and mangostin quantification

Maceration and fractionation of the mangosteen pericarp were carried out using a procedure similar to that reported by Mulia et al. [15]. Dried mangosteen pericarp powder was mixed with 96% ethanol at a ratio of 1:3 (w/v) and macerated for 14 days with continuous stirring. The fractionation of extracts obtained from maceration was performed using a mixture of ethyl acetate and water (volume ratio of 1:1) in a separating funnel. The ethyl acetate fraction was separated and solvent was evaporated using a rotary evaporator.

The uv-spectrophotometry and high-performance liquid chromatography (HPLC) were used to determine the total mangostin and the α-mangostin content, respectively. The total mangostin in the ethyl acetate fraction was quantified as α-mangostin equivalent, based on the absorbance curve of α-mangostin standard solutions measured at a wavelength of 316 nm. The α-mangostin content was determined using a Shimadzu LC 20AD HPLC equipped with a reversed-phase C18 column (250nm×4.6nm, 5μm) at maintained at 30°C, and, a UV detector set at a wavelength of 244 nm. The mobile phase consisted of 95% acetonitrile and 5% buffer (0.1% H_3_PO_4_) was used with a flow rate of 1 mL/min and elution time of 8 min [16].

### Solubility of mangosteen extract

The solubility test of the mangosteen extract in VCO, PKO and EVOO was performed by stirring the extract in oil at 500 RPM for 10 minutes at 50°C to facilitate solubilization. Mixtures of extract and oil were stored at 26–27°C for 5 days to visually observe the presence of undissolved extract in oil. Solubility data of mangosteen extract in VCO, reported by Pratiwi et al. [17], was available for comparison purposes.

### Nanoemulsion preparation

Nanoemulsions were prepared by using a high-shear stirring or high-energy (HE) and a spontaneous or low-energy (LE) emulsification methods. It was reported that a mixture of surfactants with an HLB value of 12 will provide the optimal conditions for forming stable nanoemulsion, using VCO as the oil phase [18]. In this study, a mixture of surfactant with an HLB value of 12 was prepared using Tween 80 (HLB of 15, 72.0% w/w) and Span 80 (HLB of 4.3, 28.0% w/w). Overall, two sets of nanoemulsion formulations were prepared using an HE and an LE nanoemulsification methods. The effect of composition on the stability of these nanoemulsions were studied by varying the extract to VCO ratio, water to VCO ratio, surfactant to VCO ratio, and the amount of xanthan gum added to the aqueous phase.

The composition of the nanoemulsions prepared using the HE emulsification method is given in Table 2. Firstly, the mangostin extract was mixed with the oil phase and stirred at 500 RPM and 50°C until the extract was completely dissolved. Then, the Tween 80−Span 80 surfactant mixture was added to the oil phase and stirred at 750 RPM and 50°C for 10 minutes. The oil mixture was then added to the aqueous phase gradually with a stirring speed at 750 RPM, without heating, until it was well mixed. The final step was homogenization at a speed of 8,000 RPM for 15 minutes using an Ultra Turrax homogenizer.

The composition of the nanoemulsions prepared using the LE emulsification method, modified based on the previous reports [19–23], is given in Table 3. The mangostin extract and oil phase were mixed and stirred at 500 RPM at 50°C. Then, the surfactant mixture was gradually added to the oil phase and mixed at 750 RPM for 10 minutes at 50°C. The oil phase was slowly dropped into the aqueous phase at about 2 mL/min with a stirring speed of 750 RPM and without heating until it was well mixed. The formulations included two kind of surfactant used (single and mixture), two temperature of mixing (25 and 40°C), different amount of xanthan gum added to the aqueous phase (none and 0.1% w/w), and the addition of ethanol in the oil phase.

### Physicochemical characterization and accelerated stability testing

The observation and measurement were conducted for a 28-day period. The pH, viscosity, droplet size, and zeta potential were the physical parameters observed. The data were collected on days 0, 7, 14, 21, and 28. The viscosity data were collected manually using a Cannon-Fenske pipe at 25°C and kinematic viscosity was measured in cSt units. The size, polydispersity and zeta potential of emulsion droplets were measured using a Nanoplus Particulate Systems particle size analyzer. Stability and emulsion shelf life were observed using the accelerated stability test, in which the emulsion was centrifuged at 3,800 RPM for 5 h. As many as 10 mL sample was taken for each test and observed visually for changes or separations. By measuring the height of the separated parts after centrifugation, the predicted emulsion shelf life can be calculated using the following equation:

Emulsionstability(%)=(heightofstableemulsion)/(totalheightofemulsion)×100 (1)

### Antioxidant assay of nanoemulsion and mangosteen extract

The antioxidant capacity of the mangostin extract and nanoemulsion was measured based on free radical scavenging activity with 2,2-diphenyl-1-picrylhydrazyl (DPPH). The following methods have been used by Gan and Latiff, with slight modifications [24]. Quercetin compound solution, as a standard, was prepared to obtain variations in concentration from 0.1 ppm to 500 ppm. The standard solution was added a 0.1 mM DPPH solution in ethanol, stirred, and kept in a dark room for 30 min to avoid the degradation of DPPH due to exposure to light. The absorbance of each sample was measured at a wavelength of 517 nm against ethanol as a blank/control solution. These steps were repeated on the mangostin extract and nanoemulsion samples. Free radical scavenging activity was determined using the following equation:

Scavengingactivity(%)=(Ac−As)/Ac×100 (2)

where As is the absorbance of the sample and Ac is the absorbance of the blank solution.

### Organoleptic evaluation

All samples of nanoemulsions were observed physically every week for a 4-week period. The parameters observed were color, scent, and visual phase separation.

### *In vitro* percutaneous penetration evaluation

An *in vitro* skin penetration evaluation was carried using a Franz diffusion cell and the skin of mice [25–27]. Firstly, mice skin was prepared by removing the fatty tissue and cleaned with a phosphate-buffered solution (PBS) with a pH of 7.4. The skin sample was stored in the refrigerator at a temperature of 2−4°C in PBS before usage. An evaluation was performed by placing the skin sample between the donor and receptor chambers, where the skin horn layer faced the donor chamber. The donor chamber contains the nanoemulsion sample while the receptor chamber contains the PBS. A certain amount of the nanoemulsion sample was inserted into the donor chamber, and a total of 2.5 mL of PBS was injected into the receptor. Nanoemulsion samples passing through the skin surface were accommodated in a receptor chamber that was equipped with a 600 RPM magnetic stirring bar at 37°C. The samples from the receptor chamber were taken out at a rate of 2.5 mL at 0 min, 30 min and every 1 h during the 8-h observation period. Fresh PBS was added to the receptor chamber to replace each 2.5 mL of sample and thus maintain the total volume of the PBS. The samples taken from the receptor chamber were analyzed via a UV-Vis spectrophotometer to quantify the α-mangostin that had penetrated into the skin.

## Results and discussion

### Mangosteen extract, total mangostin and α-mangostin quantification

The result of maceration and fractionation was a very thick paste of mangosteen extract as the ethyl acetate fraction with an extraction yield of 7.3% (g extract/g dry plant powder). The percentage of total mangostins in the extract, determined using UV-spectrophotometry analysis and expressed as α-mangostin equivalent, was 70.6 ± 0.6% (g total mangostin/g extract). The α-mangostin content in the extract, based on the HPLC analysis, was 44.0 ± 0.3% (g α-mangostin/g extract). The significant difference in the mangostin content between the two methods was because HPLC analysis was specific for α-mangostin only, while UV spectrophotometry was used to determine all types of mangostins in the extract that absorps UV light at 316 nm.

### Solubility of mangosteen extract in the oil phase

The solubility of the extract in three types of oil was studied. The results of the visual observation of 18 oil samples that had been kept in a dark room for five days is shown in Table 1. The presence of precipitates or undissolved extract in the oil meant that the oil phase was supersaturated. Based on visual observation, the highest solubility for the extract in the oil was as much as 1.10 mg/mL of VCO. Very tiny droplets were oberved in the oil when the concentration of the extract was increased to 1.20 mg/mL. The solubility of mangosteen extracts in the oil phase observed in this study is much higher than the reported solubility of 0.156 mg extract/mL VCO [17], probably due to the much longer solid-liquid contact time used in the present study. As the extract is much more soluble in VCO than it is in PKO and EVOO, virgin coconut oil was subsequently used as the oil phase in the preparation of the nanoemulsions. High extract to VCO ratios were to ensure a high concentration of mangostin in the nanoemulsion.

**Table 1:** Emulsion formulation and visual observation of extract solubility in the oil phase.

Emulsion #	Extract to oil ratio (mg/mL)	Oil phase		
		VCO	EVOO	PKO
1	0.29	‒	**+**	‒
2	0.33	‒	**+**	‒
3	0.40	‒	**+**	**+**
4	0.45	‒	**+**	**+**
5	0.67	‒	**+**	**+**
6	1.00	‒	**+**	**+**
7	1.10	‒	**+**	**+**
8	1.20	**+**	**+**	**+**

Precipitates in the oil phase: not observed (‒), observed (+).

### Stability of nanoemulsions prepared via the high-energy method

The nanoemulsion preparation began with dissolving the mangostin extract in VCO in the ratio selected previously. In the preparation of the nanoemulsions, surfactant was added to increase the solubility of the extract in the oil phase. Table 2 shows the stability data gathered based on visual observation for emulsions prepared by the high-energy method where concentration of extract in the VCO phase vary from 0.67 mg/mL to 1.20 mg/mL.

**Table 2:** Composition of nanoemulsions formed using the high-energy method and stability data for 28 days of visual observation.

Emulsion code*	Extract to VCO ratio (mg/mL)	% w/w					Stable up to (#day)
		Extract	VCO	Surfactant	Aqueous phase	Xanthan gum	
H11	0.67	0.014	19.4	27.7	52.9	-	3
H21	1.00	0.021	19.5	28.0	52.5	-	3
H31	1.10	0.023	19.5	27.8	52.7	-	3
H41	1.20	0.025	19.7	28.0	52.4	-	3
H12	0.67	0.011	15.0	21.5	63.5	-	2
H22	1.00	0.016	15.1	21.7	63.2	-	2
H32	1.10	0.018	15.2	21.6	63.3	-	2
H42	1.20	0.019	15.2	21.7	63.1	-	2
H13	0.67	0.008	11.4	16.3	72.3	-	28
H23	1.00	0.012	11.5	16.5	72.0	-	28
H33	1.10	0.013	11.5	16.4	72.1	-	28
H43	1.20	0.015	11.6	16.4	72.0	-	28
H1-XG	0.67	0.010	13.7	31.2	55.0	0.1	16
H2-XG	1.00	0.015	13.7	31.2	55.1	0.1	3
H3-XG	1.10	0.016	13.6	31.5	54.8	0.1	2
H4-XG	1.20	0.018	13.7	31.3	54.9	0.1	28

*: Emulsion code: high-energy emulsification method (H), extract to VCO ratio (first digit), water to oil mass ratio (second digit), and xanthan gum added as a thickening agent (XG).

Emulsions H11−H41, having the oil phase content of around 19% of the total weight, were unstable. Reducing the oil phase to 15% by weight still resulted in unstable emulsions (H12 up to H42). Further reducing the oil phase to 11.5% by weight resulted in stable nanoemulsions. As shown in Table 2, the emulsions with a composition of 11.5% (w/w) oil phase, 16.5% (w/w) surfactant, 72% (w/w) aqueous phase and extract content ranged from 0.008% to 0.015% (w/w) remained stable over 28 days of visual observation. Note that a surfactant to oil ratio of 1.4 was maintained in all of the emulsions H11−H43, similar to the results obtained in our previous study [18].

In order to ensure a high concentration of extract in the emulsion, having a large fraction of the oil phase in the emulsion is preferable. Table 2 shows the result of increasing the oil phase to 13.7%, with 31.2% surfactant, 55% aqueous phase and the addition of 0.1% xanthan gum (XG). The emulsion (H4−XG) formed was stable over 28 days of observation when using oil with extract content as high as 0.018% (w/w). With less extract in the oil phase, given the same composition of oil, aqueous phase, and surfactant, the emulsions formed were not stable. Extract to oil phase ratio of 1.20 mg/ml oil seemed to produce stable emulsions in the presence of xanthan gum.

### Stability of nanoemulsions prepared via the low-energy method

In the low-energy method, using spontaneous emulsification, the oil phase containing the extract and surfactants was titrated into the water phase at a rate of 2 ml/minute with stirring at 750 RPM. In titrating the oil phase into the water phase, the aimed was that hydrophilic substances, along with surfactants, would diffuse rapidly into the water phase [20]. Table 3 shows that emulsions L1–L4 were unstable. The surfactants used were a single surfactant (Tween 80) and a binary surfactant (Tween 80 and Span 80) with HLB values of 15 and 12, respectively. Even though a surfactant mixture with an HLB in the range of 8–15 is usually good for O/W emulsions [28], these emulsions (L1−L4) demonstrated instability when the oil phase is less than 11% (w/w).

**Table 3:** Composition of nanoemulsions formed using the low-energy method and stability data for 28 days of visual observation.

Emulsion code*	Extract to VCO ratio (mg/ml)	% w/w					Stable up to (#day)
		Extract	VCO	Surfactant	Aqueous phase	Xanthan gum/ Ethanol	
L1	1.2	0.001	1.1	2.0	96.9		1
L2	1.2	0.013	10.0	10.0	79.9		1
L3	1.2	0.001	1.1	2.0	96.9		1
L4	1.2	0.013	10.0	10.0	79.9		1
L5	1.2	0.015	11.5	16.8	71.7		28
L6	1.2	0.015	11.4	17.0	71.6		28
L5-XG	1.2	0.015	11.3	17.1	71.5	0.1	2
L6-XG	1.2	0.015	11.3	17.1	71.5	0.1	28
L7-E	1.2	0.012	9.2	13.9	58.1	18.9	20 min
L8-E	1.2	0.010	7.8	11.5	69.2	11.5	30 min

*: Emulsion code: low-energy emulsification method (L), surfactant used for L1 and L2 was Tween 80, mixed surfactants for other emulsions; oil phase in L3, L4 and L5 were as much as 1.1%, 10% and 11.5%; temperature of mixing for L6 was 40°C, 25°C for other emulsions; xanthan gum was added into the aqueous phase of L5-XG and L6-XG; ethanol was added into the oil phase of L7-E and L8-E.

The data of emulsions L5 and L6 demonstrated the effect of temperature on the stability of an emulsion. With an emulsion composition of 11% (w/w) oil phase, 17% (w/w) surfactant, and 72% (w/w) aqueous phase, at a mixing temperature of 40°C or 25°C, without xanthan gum, the nanoemulsions (L5 and L6) were stable over 28 days of observation. The addition of xanthan gum (1.0%) at a mixing temperature of 40°C (L6-XG) increased the emulsion stability but not at 25°C (L5-XG). Xanthan gum solution has pseudoplastic properties, and its viscosity decreases with increasing temperature. However, at a temperature slightly higher than 40°C, the viscosity may increase due to a conformational change in the xanthan gum molecule [29]. Because the movement of droplets of oil phase in the aqueous continuous phase is restricted by the high viscosity of the aqueous phase, this leads to an increase in the stability of emulsions [30].

It was reported that the solubility of the hydrophilic surfactant with an active hydrophobic compound in the oil phase was improved by the addition of ethanol as a co-solvent in the oil phase [30]. In order to investigate the effect of ethanol, the addition of a significant amount of ethanol into the VCO phase was performed; hence, the composition changed due to the addition of less aqueous phase. The emulsions produced with the addition of ethanol were stable only for 20−30 min, as reported in Table 3 (emulsions L7-E and L8-E). This may have been due to the different polarities of ethanol and VCO; hence, VCO is not completely soluble in ethanol. Based on the data obtained using the low-energy method of emulsification, the most stable nanoemulsion without XG consisted of oil phase (11%-w/w), surfactant (17%-w/w), and aqueous phase (72%-w/w), mixed at 25°C or 40°C. When using xanthan gum, a stable nanoemulsion can be obtained at a mixing temperature of 40°C.

### Accelerated stability testing for shelf life

An accelerated test was performed for nanoemulsions that did not show any separation and contained the highest levels of mangostin extracts, i.e., two samples of emulsion prepared via the high-energy method (H43, H4-XG) and three samples of emulsion prepared via the low-energy method (L5, L6 and L6-XG). The five samples were centrifuged at 3,800 RPM for 5 h at 25°C. The centrifugation of the emulsions under these conditions is equivalent to the effect of gravity of one year [31]. Thus, emulsions that show no phase separation after centrifugation have a shelf life at least one year. Based on the observation of creaming, the separation and rising of the oil phase in the emulsion, the shelf life was calculated using Eq (1). Table 4 shows that the H4-XG nanoemulsion, with a weight ratio of oil to surfactant of as high as 1:2.28, experienced the highest creaming and became the least stable nanoemulsion (46.7% of a year shelf life).

**Table 4:** Results of accelerated stability of nanoemulsions.

Emulsion code	Ratio of oil to surfactant (w/w)	Height ratio of separated phase	Predicted stability of emulsions (% × 1 year)
H43	1:1.42	1:7.5	86.7
H4-XG	1:2.28	4:7.5	46.7
L5	1:1.42	0.5:7.5	93.3
L6	1:1.42	0.6:7.5	92.0
L6-XG	1:1.42	0.6:7.5	92.0

Doubling the surfactant content in the emulsion (H4-XG) preparation so as to reach 31.3% of total weight (Table 2) seemed to cause the instability of the nanoemulsion. As reported by Komaiko and McClements, there is a point at which increasing the concentration of surfactant further may lead to an increase of droplet size due to high viscosity [20]. The data shown in Table 4 also indicates that the other nanoemulsion compositions were stable for slightly less than a year. The highest stability was found in emulsion L5, with a composition of 11% oil, 17% surfactant, 72% aqueous phase and a mixing temperature of 25°C, which was predicted to have a shelf life of around 93.3% of a year (11 months and 10 days). The addition of xanthan gum seemed to thicken the nanoemulsion but slightly reduce the shelf life, to around 92% of a year (11 months and 5 days). In general, the accelerated stability testing showed that the nanoemulsions produced via the high-energy are less stable than those prepared via the low-energy emulsification method.

### Physicochemical characterization of the stable nanoemulsions

The physicochemical properties of nanoemulsions listed in Table 4 were determined in terms of pH, viscosity, droplet size, zeta potential, and mangostin content after 28 days of observation.

#### Emulsion pH

**Fig 1:**
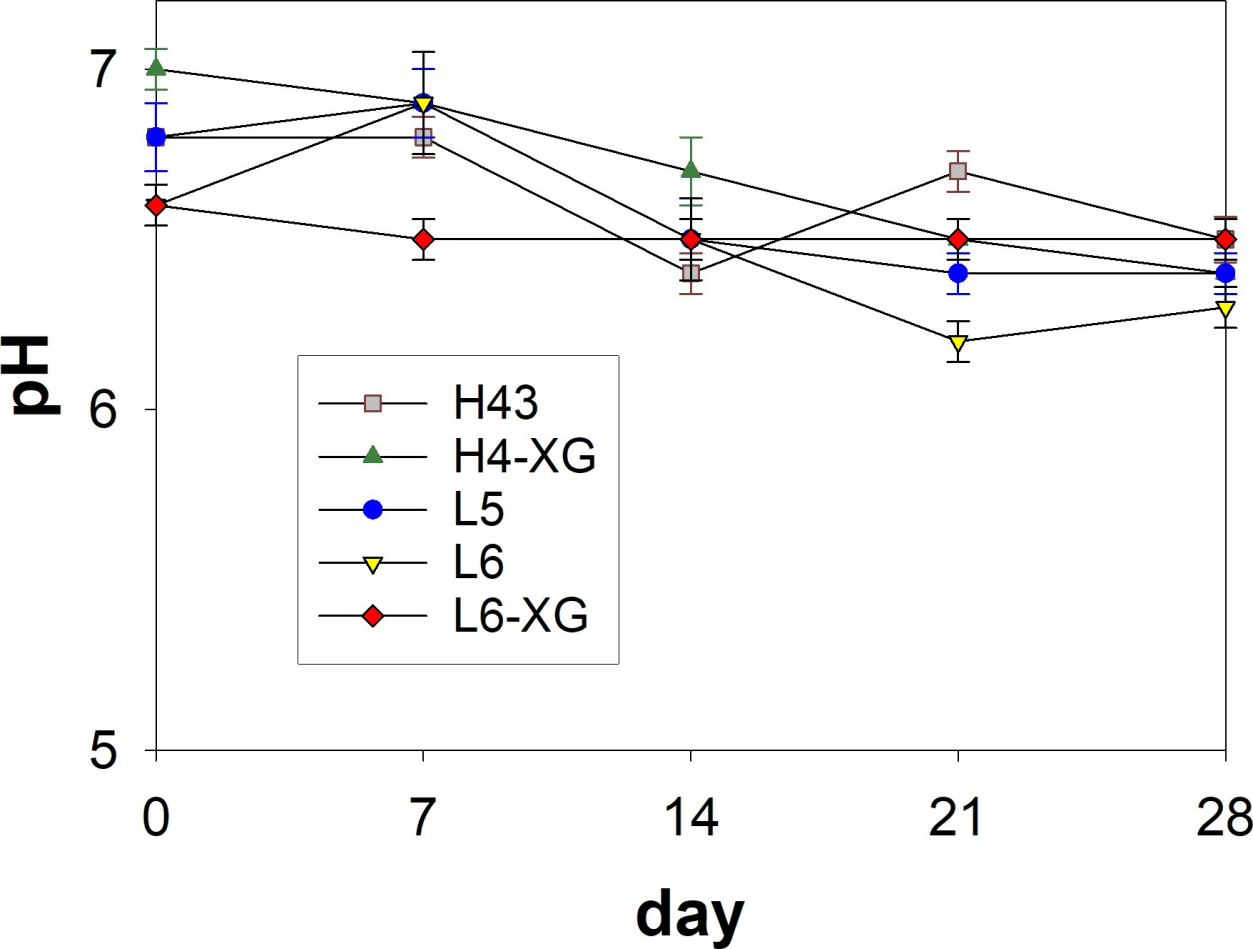
Plot of pH of nanoemulsions prepared using high (H) and low (L) energy methods as a function of storage time. Error bar represents one standard deviation.

Fig 1 shows that the five emulsion samples (H43 and H4-XG, prepared via the high-energy method; L5, L6, and L6-XG, prepared via the low-energy method) showed a narrow pH range of 6.2−6.5 over 28 days of observation. The methods of emulsion preparation seemed to have no impact on the pH values of the emulsions. According to research by Matousek et al. and Khan et al. [32, 33], and a more recent review study [34], human skin generally has a pH range from 4 to 6. For the purpose of topical applications, the pH values of these nanoemulsions are considered suitable.

#### Viscosity

**Fig 2:**
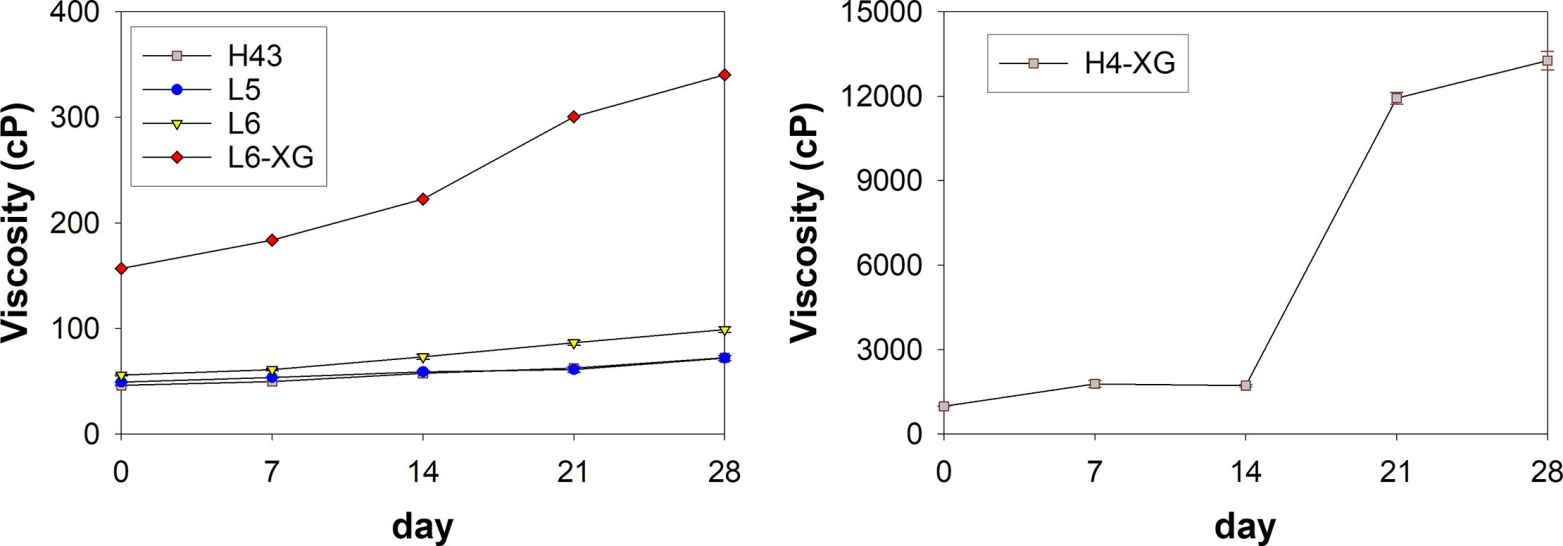
Viscosity of nanoemulsion with and without xanthan gum (XG): (a) 0−400 cP, (b) 0−14000 cP. Error bar represents one standard deviation.

Fig 2A shows that the emulsions (H43, L5, and L6) with the same weight ratio of 11:16:72 for oil: surfactant: aqueous, and without the addition of xanthan gum have quite stable viscosity levels for up to 28 days. For these emulsions, the viscosity was not affected by the emulsification methods, that is, the high-energy or low-energy methods. All samples typically showed low levels of viscosity, which ranged from 46 cP to 99 cP, with standard deviation values in the range 1–2 from triplicate data. A similar composition, though one with the addition of xanthan gum (1%) at 40°C (emulsion L6-XG), showed a viscosity that increased gradually with time, from 156 cP to 340 cP. Increasing the xanthan gum concentration in the aqueous phase may increase the viscosity of the aqueous phase because the continuous phase restricts the movement of the droplets of the oil phase, thereby preventing creaming [30] and leading stable emulsions, as indicated in Table 4.

Fig 2B shows that the H4-XG emulsion, with a weight ratio of oil:surfactant:aqueous phase of as much as 14:31:55 and with 0.1% (w/w) xanthan gum, exhibited a very drastic increase in viscosity, from 982 cP to 13,268 cP, over 28 days. Doubling the amount of surfactant and the addition of xanthan gum in emulsion H4-XG caused an increase of viscosity to more than 40-fold that of emulsion H43, which had no xanthan gum. Despite using high-shear agitation, the viscosity of H4-XG increased due to the presence of xanthan gum. This result was not in line with the viscosity of emulsions prepared via high-shear agitation without thickening agent, reported by Khan et al. and Mudoi et al. [33, 35].

At certain high surfactant concentrations, when the attractive forces between the droplets have not yet exceeded the repulsive forces between the droplets, xanthan gum may interact with surfactants, resulting in an increase in the emulsion viscosity over time. Initially, the H4-XG emulsion did not have a very high viscosity; however, over time, this value increased significantly. After three weeks of observation, the emulsion texture was close to that of an emulgel. Studies of emulsions containing herbal extracts for topical applications performed by Thomas et al. and Bujak et al. [36, 37] have reported that emulsions and creams with viscosities of 500−3,000 cP and 12,000−42,000 cP, respectively, had good spreadability. Considering this, the H4-XG emulsion in our study had a viscosity and texture resembling those of a cream for topical application. Produced using the low-energy emulsification method, the L6-XG emulsion was slightly more viscous and had better stability than the other emulsions (L5 and L6) produced using the same emulsification method (Table 4). Thus, based on the viscosity data, both H4-XG and L6-XG emulsions could be used in topical formulations.

#### Droplet size, polydispersity and zeta potential

Both droplet size and zeta potential can be used as stability parameters. The droplet size measurement is used as a stability parameter because emulsion destabilization is associated with droplet coalescence, coagulation, flocculation, and Otswald ripening [38]. In contrast, zeta potential is an electrostatic charge between the continuous phase and the dispersed phase (droplets) in the emulsion. A high zeta potential value (negative or positive) illustrates the droplets’ repulsive force [39]. The zeta potential and droplet size observations of the five stable emulsion samples over 28 days can be seen in Table 5. The emulsion preparation method has an impact on droplet size, as it is shown by the high-shear emulsions stirred at 8,000 RPM (H43 and H4-XG) having smaller droplet sizes than emulsions prepared via the spontaneous emulsification (L5, L6 and L6-XG). These five emulsion samples have been categorized as nanoemulsions as their mean droplet diameter are not much larger than 200 nm [2]. A value of PDI < 0.2 is indicative of a monodisperse droplet population.

**Table 5:** Droplet size, polydispersity and zeta potential of nanoemulsions.

Emulsion code	Droplet diameter (nm) mean ± SD (n = 3)	Polydispersity	Zeta potential (mV) mean ± SD (n = 3)
H43	237 ± 4	0.373	-52.9 ± 0.3
H4-XG	220 ± 2	0.312	-54.9 ± 0.2
L5	270 ± 3	0.365	-46.9 ± 0.2
L6	301 ± 2	0.344	-54.8 ± 0.2
L6-XG	353 ± 1	0.394	-63.7 ± 0.3

A stable colloid with moderate to excellent characteristics is known to have a zeta potential from 30 mV to 60 mV (negative or positive) or even higher [39]. The zeta potential data in Table 5 indicate that the emulsions have a high level of stability. Furthermore, the increased zeta potential of the H4-XG and L6-XG emulsions indicated the impact of the addition of xanthan gum because xanthan gum has the ability to increase a particle surface’s negative charge [40]. Based on the results in Table 5, the five emulsions met the topical dosage criteria, having a droplet size below 500 nm [41, 42]. In addition, a large negative potential, indicating the stability of hydrophobic substances, as well as the low likelihood of coagulation, was observed [39, 42].

### Mangostin encapsulation efficiency

**Fig 3:**
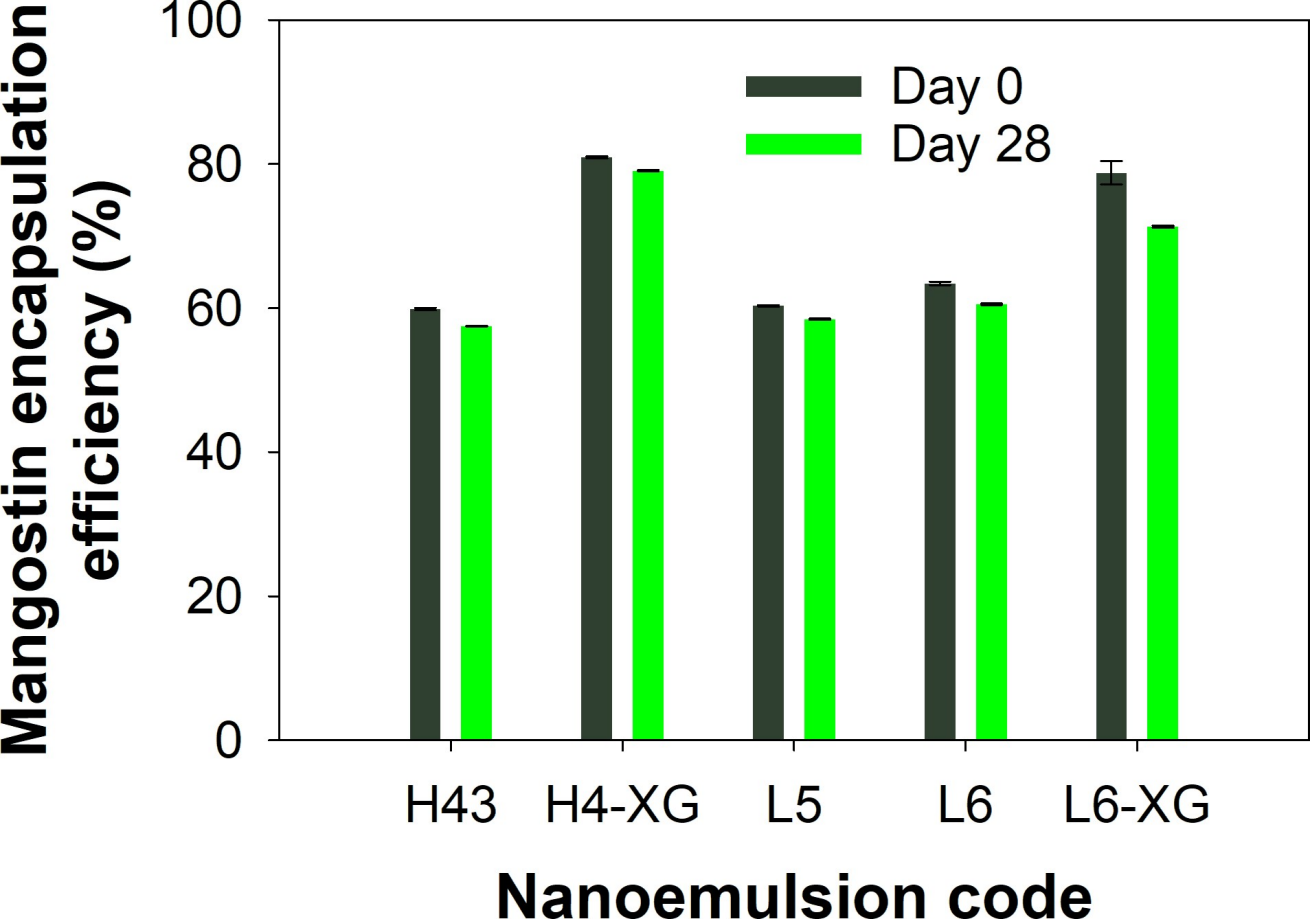
Percent encapsulation efficiency of mangostin in nanoemulsions.

The encapsulation efficiency of mangostins is a parameter indicating how much mangostins in the extract is successfully dispersed within the emulsion, defined as the mass of mangostins presents in the nanoemulsions divided by the mass of mangostins presents in the extract added to the mixture. Fig 3 shows the efficiency of mangostin encapsulation in a stable emulsion sample from the start of preparation up to 28 days. The mangostin content in each emulsion remained relatively constant, being just slightly reduced after 28 days of observation. Encapsulation efficiency ranged between 57%−81% for the five samples. The reduced mangostin content observed in each sample after 28 days may be due to several factors, such as the emulsion preparation conditions, the volume of surfactant, viscosity change, and the thickening agent. The first and the second highest encapsulation efficiencies were found in samples of nanoemulsions with xanthan gum that were prepared via the high-energy method (H4-XG) and low-energy method (L6-XG), respectively. This indicates that the addition of xanthan gum increased the encapsulation efficiency. Xanthan gum and surfactant interactions are known to stabilize dispersed droplets containing active compounds and reduce the likelihood of coagulation [43].

### Antioxidant assay of mangostin extract

**Fig 4:**
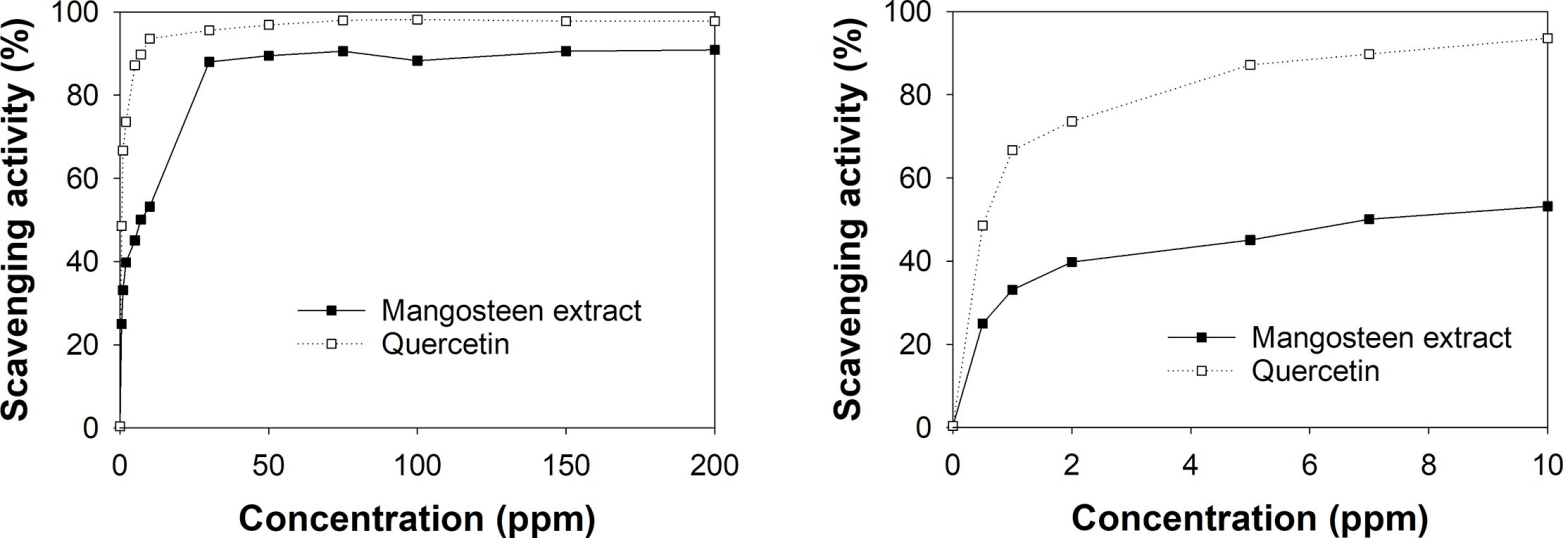
Percentage of antioxidant activity of mangosteen extract and quercetin as a standard in concentration range of (a) 0−200 ppm and (b) 0−10 ppm.

Antioxidant activity of mangostins derived from the extract of mangosteen pericarp was determined by reacting each sample with DPPH reagent and compared to quercetin as a standard compound. Fig 4A and 4B show the inhibitory or scavenging activity of the quercetin and mangosteen extract, calculated using Eq (2). Fig 4A shows that both mangosteen extract and quercetin have high antioxidant activities up to 90% and 98% scavenging activity, respectively. Fig 4B shows that the concentrations that resulted in the 50% inhibition of free radicals (IC_50_) values for the extract samples and quercetin samples were 7.0 ppm and 0.5 ppm, respectively.

The low value of IC_50_ of the extract sample indicates that the extract of mangosteen pericarp has very high antioxidant activity. An antioxidant activity test on the five emulsion samples was also conducted to determine the inhibitory activity and IC50; however, the color and absorbance of the five emulsion samples did not provide conclusive results. Using a simple calculation, the composition of the sample with 0.015% (w/w) extract in the emulsion, shown in Table 2, is equal to 150 ppm. The dilution of the nanoemulsion up to seven fold could provide an extract concentration of around 20 ppm and an inhibition activity of around 90%. Dilution up to 21 fold would provide an emulsion with an extract concentration of around 7 ppm, which is equal to the IC_50_ value of the mangosteen extract. Therefore, the emulsion samples tested contained sufficient amounts of mangostin to impart significant free radical inhibition activity.

### Percutaneous penetration (*in vitro*) evaluation

**Fig 5:**
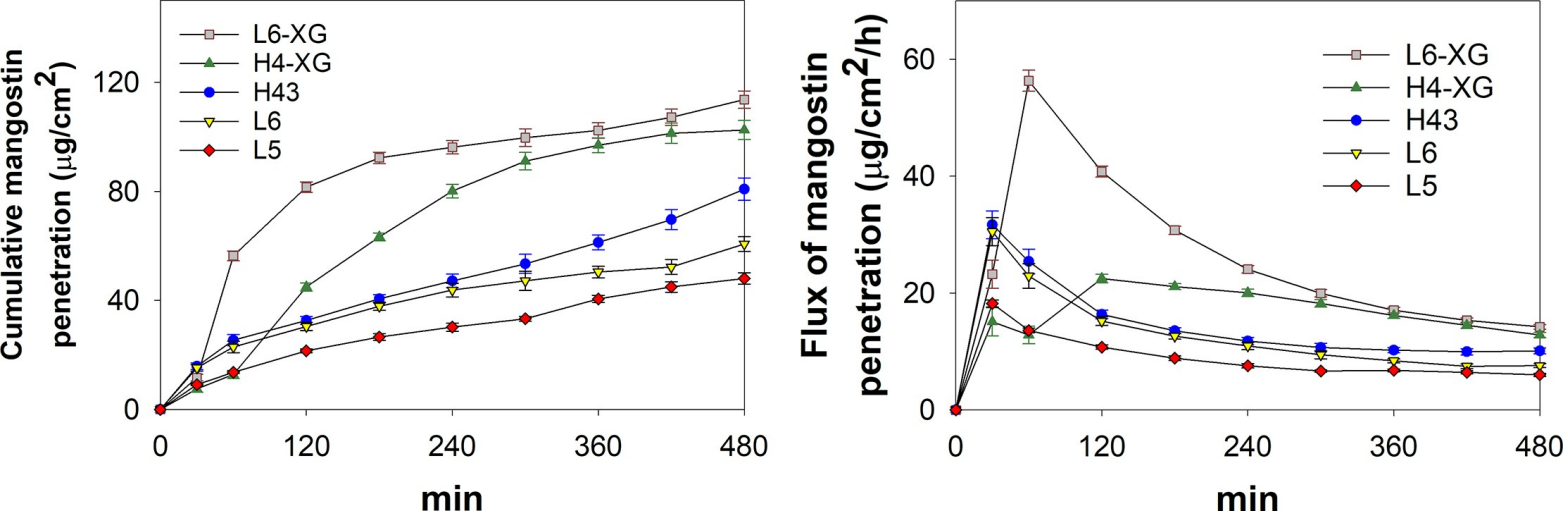
Mangostin penetration: cumulative (a) and flux (b). Error bar represents one standard deviation.

*In vitro* percutaneous penetration tests were performed to determine the amount of mangostin that could penetrate the skin membrane during an 8-hour test. Fig 5A shows that the emulsions with xanthan gum L6-XG and H4-XG provided the highest amount of mangostin penetrating the skin with values of 114 μg/cm^2^ and 102 μg/cm^2^, respectively. It seems that more active compounds penetrated into the skin due to the addition of XG, which affects the emulsion permeation rate. It is reported that the presence of XG may affect the viscosity and osmotic pressure of samples in the donor chamber of the diffusion cell [40, 44]. The amounts of mangostin that penetrated into samples without XG for nanoemulsions H43, L5, and L6 were 81 μg/cm^2^, 48 μg/cm^2^, and 61 μg/cm^2^, respectively. The release of mangostin compounds from the oil phase is affected by the solubility of mangostins in the aqueous phase and the oil-surfactant interphase because the mangostin molecule cannot penetrate from the oil phase into the skin layer directly [45]. Therefore, the mangostin penetrates the skin gradually. The release of mangostin compounds into the skin layer is also related to the size of the droplets. Droplet sizes smaller than 3 μm can be randomly distributed to the skin’s horn layer and penetrate the horn layer via three release routes, i.e., through hair follicles, sweat glands, and the stratum corneum [46]. Fig 5B shows that the maximum fluxes of five emulsion samples measured at the 30^th^ and 60^th^ minutes of observation.

The high flux at the beginning of the test indicated the rapid penetration of the emulsion through the skin layer, resulting from the mangostin concentration gradient between the donor chamber and the receptor chamber. In addition to droplet size and viscosity, the flux and amount of mangostins penetrating the skin also depend on the type, absorption ability, moisture, pores, and pH of the skin layer [37]. On the percutaneous penetration test, the emulsion sample L6-XG showed the highest mangostin penetration into the skin, and the maximum flux of this sample occurred at the 60^th^ minute of observation.

### Organoleptic evaluation

An organoleptic evaluation is a physical evaluation that aims to assess aesthetic value based on the user. Table 6 is an evaluation of five emulsion samples by 15 respondents. Based on the organoleptic evaluation, the colors and odors of all the samples were quite similar. The textures of samples with xanthan gum were certainly more viscous and creamy, with an after-feel that was soft for samples without XG but slightly sticky for sample with XG (L6-XG). The ease of removal was high for all samples except H4-XG. In general, the respondents provided highest positive responses regarding sample L6-XG as a formula for topical application.

**Table 6:** Organoleptic evaluation of nanoemulsions.

Parameter	Emulsion				
	H43	H4-XG	L5	L6	L6-XG
Texture	runny	very viscous	runny	runny	viscous
Color	milky white	ivory white	milky white	ivory white	ivory white
Odor	VCO	VCO	VCO	VCO	VCO
After-feel	soft	very sticky	soft	soft	slightly sticky
Removal	easy	oily residue	easy	easy	easy

## Conclusions

This study shows that the solubility of mangosteen extract in virgin coconut oil is 1.1 mg extract/ml oil, much higher than those in extra virgin olive oil or palm kernel oil. Surfactant concentration of around 16–17% (w/w) stabilized the nanoemulsions prepared via both the spontaneous and high-shear emulsification methods. Nanoemulsions prepared using both of the high-energy and the low-energy emulsification methods were found to be stable up to 28 days, while maintaining the highest amount of mangostin extract to oil ratio of 1.2 mg/ml. The oil droplet size (220 nm−353 nm) and zeta potential (-46.9 mV to -63.7 mV) indicate the stability these nanoemulsions. The accelerated stability test shows that the three most stable nanoemulsions were those prepared using the low-energy emulsification method with an estimated shelf life of eleven months were composed of 11% oil phase, 17% surfactant, and 72% aqueous phase. The *in vitro* percutaneous penetration test indicates that the nanoemulsion formulated with xanthan gum as the thickening agent and mixed at a temperature of 40°C showed the highest amount of mangostin penetrating into the skin, with the maximum flux occurring after one hour of application. These results indicate that VCO nanoemulsions prepared using low-energy emulsification method with xanthan gum as a thickening agent are prospective vehicles for the topical delivery of mangosteen extract.

## Supporting information

S1 Table Scavenging activity of mangosteen extract and quercetin. (DOCX)

S2 Table Cumulative mangostin penetration data. (DOCX)
