# Supplementary material for: Nanoemulsions containing Garcinia mangostana L. pericarp extract for topical applications: Development, characterization, and in vitro percutaneous penetration assay
Source: PLoS One. 2021 Dec 23;16(12):e0261792. doi: 10.1371/journal.pone.0261792 (PMC8700051; doi:10.1371/journal.pone.0261792)
Supplement: S1 Table — (DOCX) [file pone.0261792.s001.docx]

## S1 Table: Scavenging activity of mangosteen extract and quercetin.

| Concentration (ppm) | Scavenging activity (%) | |
| --- | --- | --- |
|  | Mangostin extract | Quercetin |
| 0 | 0 | 0 |
| 0.5 | 25.0 | 48.6 |
| 1 | 33.1 | 66.7 |
| 2 | 39.8 | 73.6 |
| 5 | 45.1 | 87.2 |
| 7 | 50.1 | 89.8 |
| 10 | 53.2 | 93.6 |
| 30 | 88.0 | 95.6 |
| 50 | 89.5 | 96.9 |
| 75 | 90.6 | 98.0 |
| 100 | 88.3 | 98.2 |
| 150 | 90.6 | 97.8 |
| 200 | 90.9 | 97.8 |
