# Supplementary material for: Nanoemulsions containing Garcinia mangostana L. pericarp extract for topical applications: Development, characterization, and in vitro percutaneous penetration assay
Source: PLoS One. 2021 Dec 23;16(12):e0261792. doi: 10.1371/journal.pone.0261792 (PMC8700051; doi:10.1371/journal.pone.0261792)
Supplement: S2 Table — (DOCX) [file pone.0261792.s002.docx]

## S2 Table: Cumulative mangostin penetration data.

| time (min) | Nanoemulsion | | | | | | | | | |
| --- | --- | --- | --- | --- | --- | --- | --- | --- | --- | --- |
|  | L6-XG | | H4-XG | | H43 | | L6 | | L5 | |
|  | CMP | SD | CMP | SD | CMP | SD | CMP | SD | CMP | SD |
| 0 | 0 | 0 | 0 | 0 | 0 | 0 | 0 | 0 | 0 | 0 |
| 30 | 11.6 | 1.2 | 7.5 | 1.2 | 15.8 | 1.2 | 15.3 | 1.2 | 9.1 | 0.3 |
| 60 | 56.3 | 1.8 | 12.8 | 1.5 | 25.4 | 2.1 | 22.9 | 2.1 | 13.6 | 0.4 |
| 120 | 81.6 | 1.9 | 44.9 | 1.5 | 32.7 | 1.5 | 30.4 | 1.5 | 21.5 | 0.7 |
| 180 | 92.3 | 2.1 | 63.3 | 1.5 | 40.6 | 1.5 | 37.8 | 1.5 | 26.5 | 1.2 |
| 240 | 96.2 | 2.5 | 80.1 | 2.5 | 47.1 | 2.5 | 43.8 | 2.5 | 30.2 | 1.5 |
| 300 | 99.7 | 3.2 | 91.1 | 3.2 | 53.4 | 3.5 | 47.2 | 3.5 | 33.2 | 0.9 |
| 360 | 102.4 | 2.8 | 96.9 | 2.7 | 61.2 | 2.7 | 50.4 | 2.1 | 40.5 | 1.3 |
| 420 | 107.2 | 3.0 | 101.3 | 3.7 | 69.7 | 3.7 | 52.2 | 2.7 | 44.9 | 1.9 |
| 480 | 113.7 | 3.2 | 102.5 | 3.5 | 80.8 | 4.1 | 60.7 | 2.7 | 48.0 | 2.1 |
| CMP: mean cumulative mangostin penetration in μg/cm^2^ (n=3)  SD: standard deviation (n=3) | | | | | | | | | | |
